# *Varroa destructor* shapes the unique viral landscapes of the honey bee populations of the Azores archipelago

**DOI:** 10.1371/journal.ppat.1012337

**Published:** 2024-07-03

**Authors:** Ana R. Lopes, Matthew Low, Raquel Martín-Hernández, Joachim R. de Miranda, M. Alice Pinto

**Affiliations:** 1 Centro de Investigação de Montanha (CIMO), Instituto Politécnico de Bragança, Campus de Santa Apolónia, Bragança, Portugal; 2 Laboratório Associado para a Sustentabilidade e Tecnologia em Regiões de Montanha (SusTEC), Instituto Politécnico de Bragança, Campus de Santa Apolónia, Bragança, Portugal; 3 REQUIMTE-LAQV, Faculdade de Farmácia, Universidade do Porto, Rua de Jorge Viterbo Ferreira, Porto, Portugal; 4 Department of Ecology, Swedish University of Agricultural Sciences, Uppsala, Sweden; 5 Centro de Investigación Apícola y Agroambiental (CIAPA), IRIAF, Instituto Regional de Investigación y Desarrollo Agroalimentario y Forestal, Marchamalo, Spain; Boston College, UNITED STATES

## Abstract

The worldwide dispersal of the ectoparasitic mite *Varroa destructor* from its Asian origins has fundamentally transformed the relationship of the honey bee (*Apis mellifera*) with several of its viruses, via changes in transmission and/or host immunosuppression. The extent to which honey bee-virus relationships change after *Varroa* invasion is poorly understood for most viruses, in part because there are few places in the world with several geographically close but completely isolated honey bee populations that either have, or have not, been exposed long-term to *Varroa*, allowing for separate ecological, epidemiological, and adaptive relationships to develop between honey bees and their viruses, in relation to the mite’s presence or absence. The Azores is one such place, as it contains islands with and without the mite. Here, we combined qPCR with meta-amplicon deep sequencing to uncover the relationship between *Varroa* presence, and the prevalence, load, diversity, and phylogeographic structure of eight honey bee viruses screened across the archipelago. Four viruses were not detected on any island (ABPV-Acute bee paralysis virus, KBV-Kashmir bee virus, IAPV-Israeli acute bee paralysis virus, BeeMLV-Bee macula-like virus); one (SBV-Sacbrood virus) was detected only on mite-infested islands; one (CBPV-Chronic bee paralysis virus) occurred on some islands, and two (BQCV-Black queen cell virus, LSV-Lake Sinai virus,) were present on every single island. This multi-virus screening builds upon a parallel survey of Deformed wing virus (DWV) strains that uncovered a remarkably heterogeneous viral landscape featuring *Varroa*-infested islands dominated by DWV-A and -B, *Varroa*-free islands naïve to DWV, and a refuge of the rare DWV-C dominating the easternmost *Varroa*-free islands. While all four detected viruses investigated here were affected by *Varroa* for one or two parameters (usually prevalence and/or the Richness component of ASV diversity), the strongest effect was observed for the multi-strain LSV. *Varroa* unambiguously led to elevated prevalence, load, and diversity (Richness and Shannon Index) of LSV, with these results largely shaped by LSV-2, a major LSV strain. Unprecedented insights into the mite-virus relationship were further gained from implementing a phylogeographic approach. In addition to enabling the identification of a novel LSV strain that dominated the unique viral landscape of the easternmost islands, this approach, in combination with the recovered diversity patterns, strongly suggests that *Varroa* is driving the evolutionary change of LSV in the Azores. This study greatly advances the current understanding of the effect of *Varroa* on the epidemiology and adaptive evolution of these less-studied viruses, whose relationship with *Varroa* has thus far been poorly defined.

## Author summary

Honey bees are plagued by many enemies, and the *Varroa* mite is one of the most important of these. *Varroa* hurts bees by feeding on their haemolymph, but more importantly, by facilitating the transmission and development of many viruses. The impact of *Varroa* on most honey bee viruses remains poorly understood. Here, we capitalized on the exceptional Azores setting, which contains islands with and without *Varroa*, to gain unprecedented insights into the complex mite-virus interactions. We uncovered a very heterogenous viral landscape, with one virus (SBV) occurring only on mite-infested islands, two (CBPV and DWV) on some islands, and two (BQCV and LSV) on every single island. While *Varroa* influenced the prevalence and/or diversity of all four viruses, its strongest effect was observed for LSV, with the mite leading to elevated LSV prevalence, loads, and diversity (number of variants and their relative abundance). Furthermore, we discovered a novel LSV strain and showed for the first time that the epidemiology of LSV-2, a major strain of LSV, is unambiguously linked to the presence of *Varroa*. Our findings not only deepen current scientific understanding of the mite-virus relationships but are also of value for assisting veterinary authorities in decision-making regarding the movement of bees across territories.

## Introduction

Honey bee (*Apis mellifera* L.) colonies around the world suffer heavy losses every year, with negative consequences for honey production, crop pollination and food production [1–3]. While there is a wealth of interacting abiotic and biotic factors underlying these losses, viruses are recognized as important players in colony health [4,5]. Over 50 viruses have been identified in honey bees [6] and many are shared with solitary and social wild bees [7]. The great majority are isometric particles containing single-stranded positive sense RNA (+ssRNA) genomes, with those belonging to the *Dicistroviridae*, *Iflaviridae*, and *Sinhaliviridae* comprising the most detected or economically important viruses in honey bees. Important dicistroviruses include Black queen cell virus (BQCV; *Triatovirus nigereginacellulae*), Acute bee paralysis virus (ABPV; *Aparavirus apisacutum*), Kashmir bee virus (KBV; *Aparavirus kashmirense*), and Israeli acute bee paralysis virus (IAPV; *Aparavirus israelense*). Important iflaviruses include Slow bee paralysis virus (SBPV; *Iflavirus apistardum*), Sacbrood virus (SBV; *Iflavirus sacbroodi*), and particularly Deformed wing virus (DWV; *Iflavirus aladeformis*). *Sinhaliviridae* is a recently created virus family containing only a single species, the common honey bee virus Lake Sinai virus (LSV) [8]. LSV was first described in the early 2000’s in migratory collapsing colonies from sites close to Lake Sinai, in the USA [9], although the two main variants (LSV-1 and LSV-2) are suspected to have been known previously as Bee virus X (BVX) and Bee virus Y (BVY), due to similarities in particle size, shape, genome and infection characteristics [10]. Since then, eight unique LSV strains have been described around the world, consistent with LSV’s high mutation rate [11,12]. DWV is another multi-strain virus, although it comprises only four master variants, including the common DWV-A [13] and DWV-B [14] variants, the rare DWV-C [15] variant, and the most likely extinct DWV-D variant [16]. Finally, another economically important virus is Chronic bee paralysis virus (CBPV), which was one of the first honey bee viruses to be characterized [17] and probably the first (viral) disease recognised by beekeepers since antiquity [18].

These viruses can inflict clinical signs on the honey bees that are recognisable by beekeepers, such as deformed wings (DWV), sacbrood pupae (SBV), paralysis and denuded (black) body (CBPV), or dead queen larvae whose decaying corpses stain their wax cells black (BQCV). However, they can also remain unnoticed in apparently healthy colonies [19,20]. Indeed, adult honey bees can host BQCV, SBV, or LSV without any observable symptoms [10]. Moreover, most of the viruses can persist in the colony as covert infections until other stressors disrupt the host-virus equilibrium [21–23]. One such important stressor is the ectoparasitic mite *Varroa destructor* (hereafter *Varroa*), which feeds on haemolymph and fat body tissue of honey bee pupae and adults [24–26], thereby causing physiological, developmental, and behavioural changes in the infested individuals, with negative consequences for the health of the colony [27–30]. Not only does *Varroa* inflict direct injury, but it offers an additional transmission route for several viruses, including the recently discovered *Tymoviridae* Bee Macula-like virus (BeeMLV; [31,32]) and, more importantly, the DWV and AKI (ABPV, IAPV, and KBV) complex viruses [4,33–36], which have been associated with elevated winter colony losses [37–39] and colony mortality [40–42]. In the absence of *Varroa*, oral-faecal transmission emerges as the primary route for most virus dissemination, with honey bees acquiring viral particles during their cleaning duties within the hive, and transmitting to other bees through trophallaxis and the feeding of larvae [7].

Consistent with the acquisition of this novel, varroa-mediated viral transmission route is the rise in prevalence and/or loads observed for DWV and KBV soon after the arrival of *Varroa* [43–46]. While this is expected when viruses adapt to a potent vector such as *Varroa*, AKI surveys in Hawaii [47] and DWV surveys in the Solomon Islands [48], Fernando de Noronha [49], and the Azores [46] found no impact of the mite on the prevalence and/or loads of these viruses. Other important honey bee viruses, such as BQCV, SBV, LSV, and CBPV, for which mite-borne transmission is uncertain [6,7,10,32], have been associated indirectly with the invasion of *Varroa* [43–45]. However, again, the impact of *Varroa* on these viruses was not consistent among studies [43–45,50], suggesting further investigations are needed for a better understanding of its role in shaping viral landscapes.

While several studies have investigated the effect of *Varroa* on viral prevalence and loads [43–45,47,51], the extent and direction in which the mite can alter viral diversity at colony and population levels have been largely overlooked [47]. Understanding how *Varroa* modulates viral diversity is an important endeavour because many of these viruses were largely benign in honey bees prior to the worldwide spread of varroa, and colony losses have increased substantially afterwards [23,52]. This is attributed to the mite’s capacity for not only transmitting multiple viruses [6,7] but also for increasing the virulence of some viruses [34,53] and selecting for specific strains [47,54]. The best documented example comes from DWV, whereby DWV-A has been largely replaced by the more virulent DWV-B [55], and this evolutionary shift can explain the dramatic decrease in diversity following the mite’s arrival to Hawaii [47,51]. However, the invasion of *Varroa* does not always lead to reduced viral diversity, as observed for DWV in the Azores [46] and in laboratory studies [54].

*Varroa* was originally a parasite of the Eastern honey bee *Apis cerana*, which rapidly spread worldwide after a host shift to *Apis mellifera* in the middle of the 20^th^ century [29]. *Varroa* is now present on all continents where honey bees thrive, including Australia, where it entered in 2022 [56]. There are only a few places in the world that remain naïve to *Varroa*, including on six of the nine islands of the Azores [57]. This remote Atlantic archipelago offers a rare opportunity to investigate the intricate dynamics between *Varroa* and honey bee viruses.

Herein, we employed real-time quantitative PCR and meta-amplicon deep sequencing to provide a comprehensive view of the honey bee viral landscape in the Azores and how it changed following the arrival of *Varroa* onto three islands in the early 2000’s. Our goal was two-fold. First, we sought to complement a previous molecular survey focusing on DWV [46] by screening eight additional viruses, including some known to be transmitted by *Varroa* (BeeMLV, ABPV, KBV, and IAPV; [6,31]), some that have been associated with *Varroa* invasion (BQCV, SBV, and CBPV; [43–45]), and some that have been detected in collapsed or unhealthy colonies (LSV; [11,58–61]). Together with the DWV study, this is the first molecular screening of honey bee viruses in the Azores. We expect our findings to aid the local veterinary authorities in devising a plan for honey bee and hive product exchange among islands. Second, we sought to expand the current knowledge on the role that *Varroa* has played in shaping the honey bee virus landscape beyond what is known for DWV, the primary *Varroa*-associated virus. *Varroa* can affect honey bee viral landscapes directly, *i*.*e*. when it acts as a vector, or indirectly when it reduces honey bee immunocompetence [30], or promotes interactions between co-infecting viruses, thus exacerbating their individual effects [29,62]. These mechanisms facilitate virus replication, and thus also intra- and inter-colonial dissemination, in which case elevated prevalence, co-prevalence, loads, and diversity of *Varroa*-associated viruses would be expected upon *Varroa* invasion of previously *Varroa*-free regions.

## Results

### Effect of *Varroa* on viral prevalence

**Fig 1:**
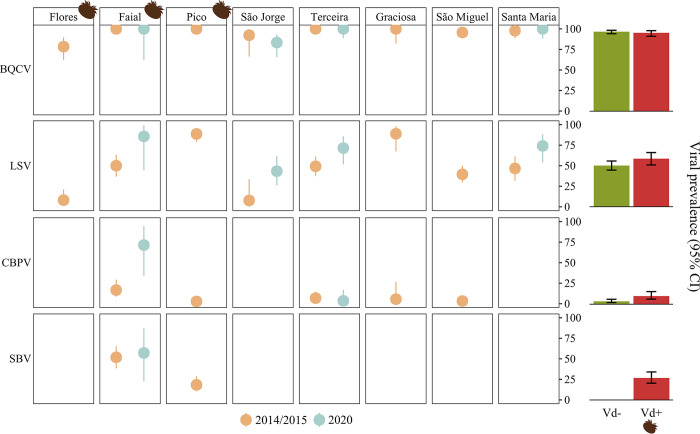
Viral prevalence (%), and estimated 95% confidence intervals, across the Azores and sampling periods (2014/2015 and 2020). Results are shown for each island individually and for the combined islands that are positive (Vd+, red bar) or negative (Vd-, green bar) to *Varroa*. Faial, São Jorge, Terceira, and Santa Maria were sampled in both sampling periods. BQCV- Black queen cell virus; LSV- Lake Sinai virus; CBPV- Chronic bee paralysis virus; SBV- Sacbrood virus. *Varroa*-invaded islands (Flores in 2000, Pico in 2001, and Faial in 2008) are denoted by the varroa icon (created by www.biorender.com).

Viral prevalence was determined by quantifying the number of colonies testing positive for each virus on each island. Viral RNA was detected in the majority of the colonies (481, 97.4%), with just 13 colonies (São Miguel = 2, Flores = 8, São Jorge = 3; 2.6%) testing negative for every surveyed virus. The three viruses of the AKI complex (ABPV, KBV, and IAPV) and BeeMLV were not detected in any colony or sampling period. This stands in stark contrast to BQCV and LSV, which occurred on every single island in both sampling periods. Across all islands and sampling periods, the highest viral prevalence was observed for BQCV (96.2%, CI 94.1–97.7%) and LSV (53%, CI 48.6–57.5%). These ranged from 78.4% (Flores) to 100% (Faial, Pico, Terceira, and Graciosa) for BQCV and 7.7% (Flores) to 88.9% (Graciosa) for LSV (Fig 1). Notably, in contrast to BQCV or the other detected viruses, which mostly maintained a stable prevalence over time, the proportion of LSV-infected colonies showed a consistent increase in 2020 compared to the preceding sampling period across all re-sampled islands. The Vd- island of São Jorge witnessed the most substantial LSV increase, from 7.7% (CI 0.4–33.7%) to 43.3% (CI 26.0–62.0%).

**Fig 2:**
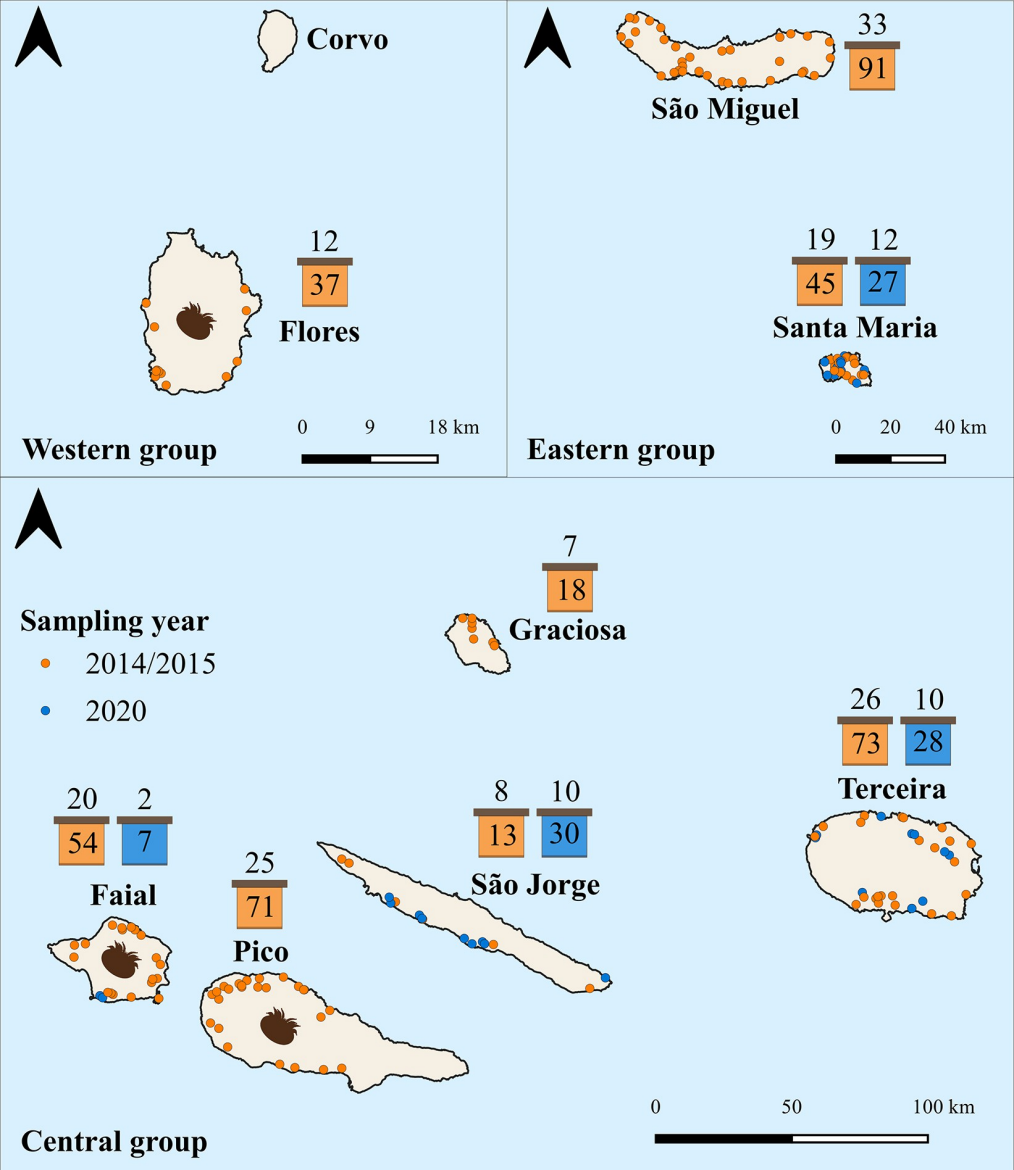
Distribution of the 150 apiaries sampled in 2014/2015 (orange) and 92 sampled in 2020 (blue). The number of colonies for each island is shown inside the hive icon and the number of apiaries is shown on top. *Varroa*-invaded islands are denoted by the varroa icon (created by www.biorender.com). The base map file of the Azores was obtained from www.diva-gis.org.

CBPV was detected on the Vd+ islands Pico (2.8%, CI 0.5–9.4%) and Faial (16.7%, CI 8.7–29.3%), but not on Flores. It was also detected on the Vd- islands Terceira (6.8%, CI 2.7–14.8), Graciosa (5.6%, CI 0.3–26.6%), and São Miguel (3.3%, CI 0.9–9.0%), but not on São Jorge and Santa Maria. Of note was the sharp rise in CBPV prevalence observed on Faial, from 16.7% in 2014/2015 to 71.4% (CI 34.1–94.7%) in 2020. However, this observation should be interpreted cautiously due to the limited geographical coverage and small sample size on this island in 2020 (Fig 2). Finally, SBV was only detected on Faial, with 51.9% (CI 38.5–65.1%) of colonies testing positive in 2014/2015, and 57.1% (CI 22.5–87.1%) testing positive in 2020, and on Pico, with 18.3% (CI 10.6–28.8%) of the colonies testing positive in 2014/2015. In summary, all four viruses were present on the Vd+ islands Pico and Faial, in contrast with the other Vd+ island Flores, which was seemingly devoid of CBPV and SBV (Fig 1).

Prevalence was then calculated for two groups: one including all the colonies from Vd+ islands and another including all the colonies from Vd- islands (bar plots on Fig 1). The prevalence of LSV, CBPV, and SBV, but not BQCV, was higher on Vd+ islands than on Vd- islands, and this finding was confirmed by Bayesian hierarchical modelling (Table 1). The strongest size effect was obtained for SBV, with *Varroa* leading to a mean increase in prevalence of 23.89 ± 17.41%, supported by a posterior probability of 100% that *Varroa* increased SBV prevalence (Table 1). For LSV, the presence of the mite also increased mean viral prevalence (19.52 ± 9.52%) with a high probability that Vd+ islands had a higher LSV prevalence than Vd- islands (Pr_(Vd+>Vd-)_ = 97.6%). While *Varroa* also had a relatively significant effect on CBPV prevalence (Pr_(Vd+>Vd-)_ = 91.6%), the mean increase was below 1% and largely influenced by the limited 2020 survey on Faial (Fig 1).

**Table 1:** Estimates of virus prevalence (%), co-prevalence (%), number of co-prevalent viruses (Nr. viruses), diversity (Richness and Shannon-Wiener index), and log_10_-loads (copies/bee) for honey bee colonies on *Varroa-*positive islands (Vd+) *versus Varroa*-negative (Vd-) islands. The estimates are the mean ± standard deviation of the posterior distributions generated by Bayesian hierarchical GLMM models that account for apiary and sampling-year effects (random and fixed, respectively). Effect size is the mean difference between Vd+ and Vd- islands. Pr_(Vd+ > Vd-)_ is the probability that this difference results in elevated estimates on Vd+ islands.

***Prevalence***				
	Vd-	Vd+	Effect size	Pr_(Vd+ > Vd-)_
**BQCV**	99.93 ± 0.30	99.97 ± 0.11	-0.04 ± 0.25	28.5%
**LSV**	55.49 ± 6.99	75.01 ± 8.25	19.52 ± 9.52	97.6%
**CBPV**	0.06 ± 0.12	0.37 ± 0.69	0.31 ± 0.63	91.6%
**SBV** ^**a**^	0.001 ± 0.01	23.89 ± 17.41	23.89 ± 17.41	100%
**Co-Prevalence**	64.37 ± 5.71	90.52 ± 3.71	26.15 ± 5.90	100%
**Nr. viruses**	1.74 ± 0.21	2.15 ± 0.12	0.41 ± 0.13	99.3%
***Richness (S)***				
**BQCV**	7.25 ± 0.28	9.42 ± 0.55	2.17 ± 0.54	100%
**LSV**	13.57 ± 0.50	16.64 ± 0.87	3.06 ± 0.93	99.9%
**CBPV**	2.20 ± 0.91	3.21 ± 0.93	1.01 ± 1.19	86.9%
***Shannon-Wiener index (H)***				
**BQCV**	0.38 ± 0.04	0.38 ± 0.05	0.00 ± 0.03	48.1%
**LSV**	0.41 ± 0.03	0.48 ± 0.04	0.07 ± 0.03	98.7%
**CBPV**	0.93 ± 0.64	1.05 ± 0.66	0.13 ± 0.40	68.2%
***Viral load***				
**BQCV**	6.77 ± 0.10	6.96 ± 0.15	0.18 ± 0.16	90.8%
**LSV**	7.50 ± 0.15	7.63 ± 0.22	0.14 ± 0.23	71.1%
**LSV-2**	5.49 ± 0.49	7.97 ± 0.36	2.48 ± 0.68	100%
**CBPV**	7.01 ± 1.06	6.52 ± 0.93	-0.49 ± 1.27	40.1%

^a^ *Varroa* effect on SBV viral loads and diversity could not be assessed because this virus was only detected on Vd+ islands.

### Effect of *Varroa* on viral co-prevalence

**Fig 3:**
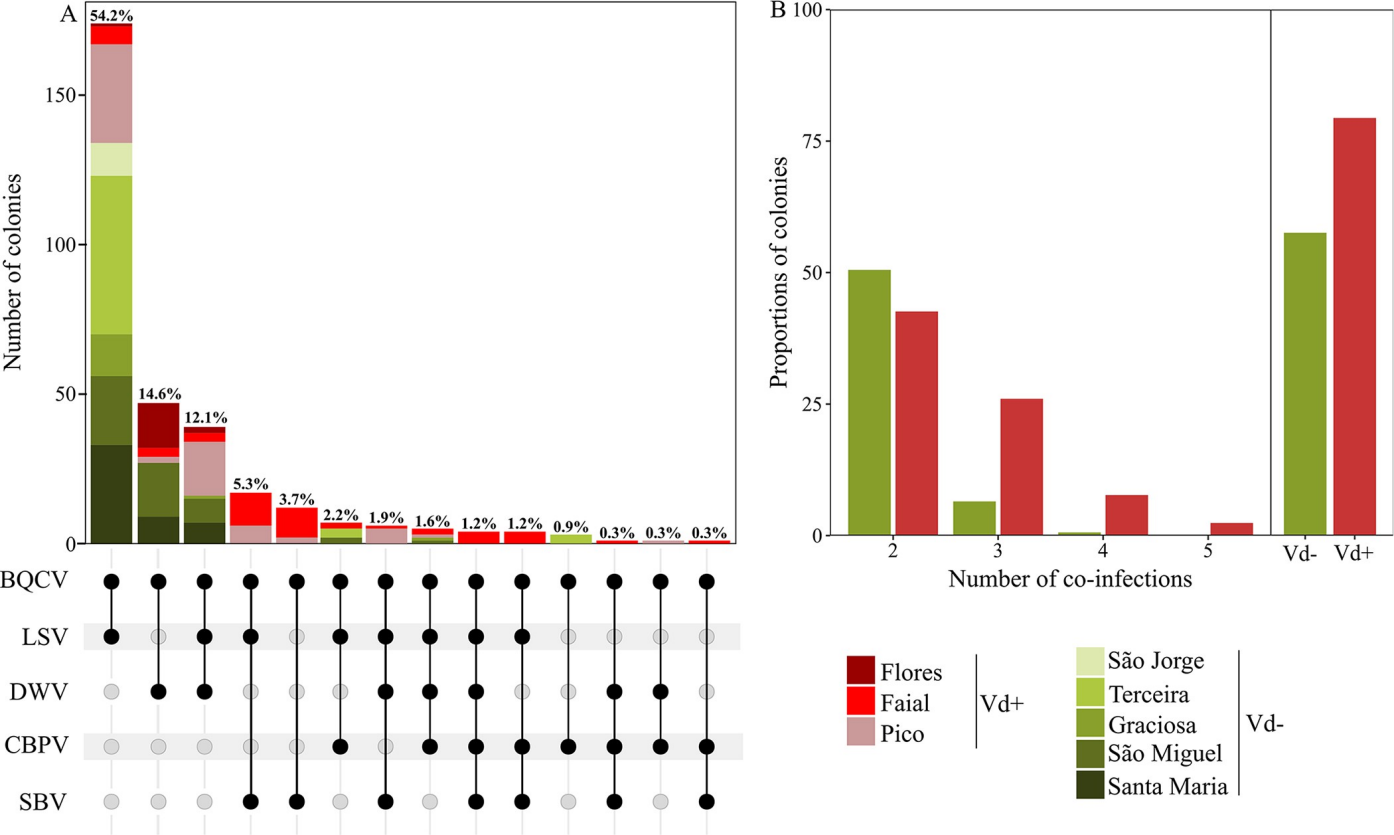
(A) Viral co-prevalence in the colonies that tested positive for at least two of the 10 screened viruses in the Azores; DWV data from [46]. (B) Proportion of colonies with 2 to 5 viruses (left) and with ≥ 2 viruses (right) on *Varroa*-positive (Vd+: red) and *Varroa* -negative (Vd-: green) islands. The red gradient represents the Vd+ islands whereas the green gradient represents the Vd- islands. The values at the top of the bars represent the percentage of the colonies with co-infections. BQCV- Black queen cell virus; LSV- Lake Sinai virus; DWV- Deformed wing virus; CBPV- Chronic bee paralysis virus; SBV- Sacbrood virus.

Of the 488 colony samples that tested positive for at least one of the surveyed viruses (including the DWV reported separately [46]), 167 samples (34.2%) were infected by a single virus, including 154 (31.6%) by BQCV, 7 (1.4%) by DWV, and 6 (1.2%) by LSV. However, the great majority of samples (321, 65.8%) were co-infected with two or more viruses (Fig 3A). The most frequent combination comprised BQCV-LSV (174 colonies, 54.2%), followed by BQCV-DWV (47 colonies, 14.6%), and BQCV-LSV-DWV (39 colonies, 12.1%). Although rare, there were also colonies co-infected by all five viruses (4, 0.8%), and these originated from the Vd+ island of Faial. On the Vd- islands, the number of co-prevalent viruses varied between two (São Jorge) and four (São Miguel).

Fig 3B depicts the viral co-prevalence for Vd+ and Vd- islands. There was a higher proportion of colonies co-infected by two viruses on Vd- than on Vd+ islands. However, the opposite trend was observed when the number of viruses was higher than two. When combining all the colonies, it became evident that co-prevalence was elevated in the presence of *Varroa*, with 79.3% of the colonies from Vd+ islands hosting multiple infections as compared to 57.5% of colonies from Vd- islands. This finding was supported by the Bayesian modelling, which estimated an increase in the mean co-prevalence of 26.15 ± 5.90% on Vd+ islands, with a posterior probability of 100% that *Varroa* increased viral co-prevalence (Table 1). Moreover, the presence of *Varroa* also led to an elevated number of co-infecting viruses (Pr_(Vd+>Vd-)_ = 99.3).

### Effect of *Varroa* on viral diversity

**Fig 4:**
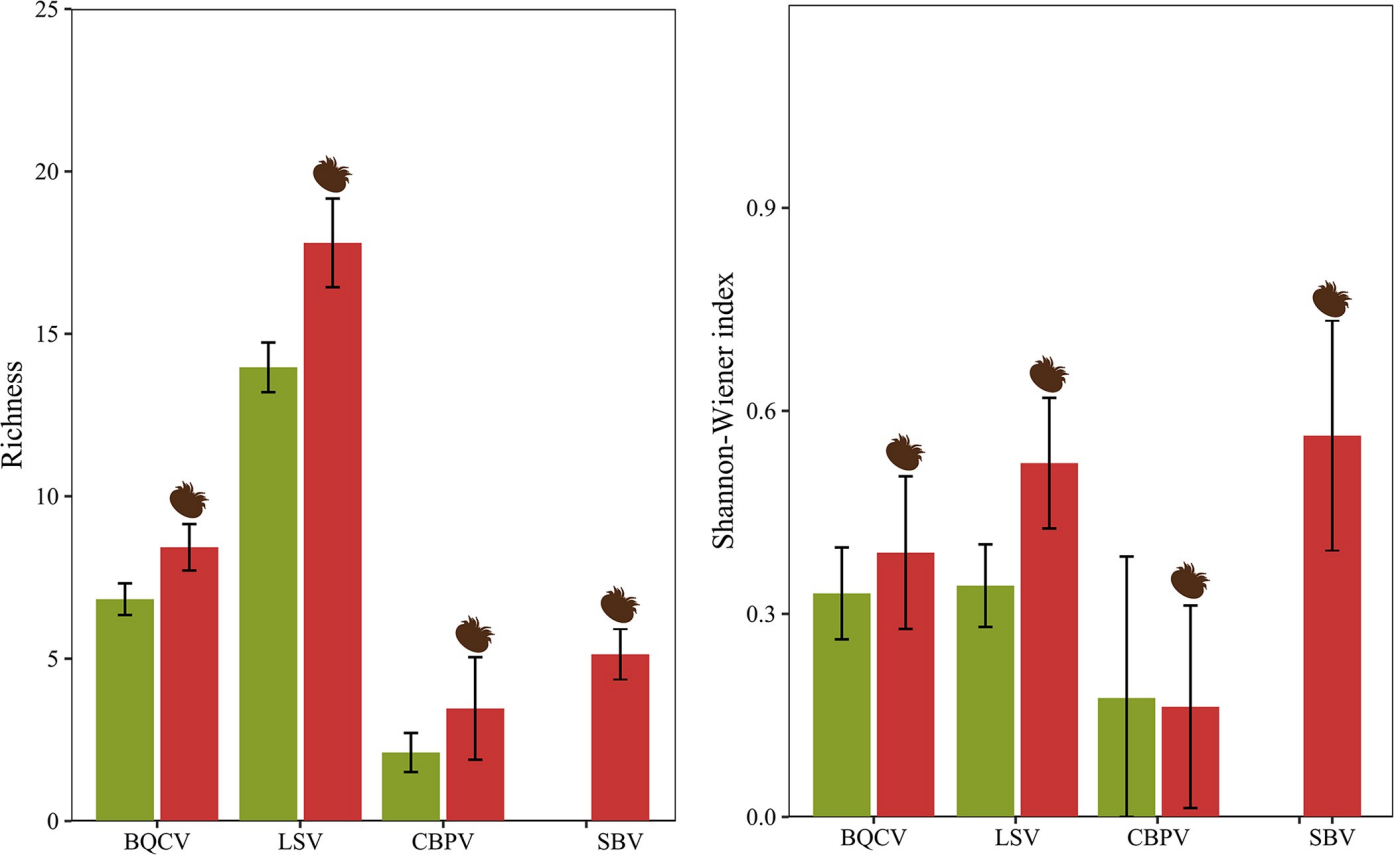
Mean (and 95% confidence interval) diversity, as expressed by Richness and Shannon-Wiener index diversity patterns inferred from the total amplicon sequence variants (ASVs) for BQCV- Black queen cell virus, LSV- Lake Sinai virus, CBPV- Chronic bee paralysis virus, and SBV- Sacbrood virus. The group of islands where *Varroa* is absent (green bar) is compared with that where *Varroa* is present (red bar). Each bar represents the mean diversity and 95% confidence interval. Varroa icon created by created by www.biorender.com.

To further unveil the effect of *Varroa* on viral landscapes, colonies testing positive for BQCV, LSV, SBV, and CBPV underwent high-throughput sequencing of the RT-PCR amplicons produced by the survey. This approach enabled retrieval of the full diversity spectrum for each virus in the Azores, here summarized by Richness (S, total number of amplicon sequence variants, ASVs) and Shannon-Wiener index (H, combining S with the relative abundance of each ASV, *i*.*e*. the ‘Evenness’ of the distribution of the ASVs). The highest diversity was observed for LSV (S = 506; H = 3.45), followed by BQCV (S = 167; H = 2.18) or SBV (S = 78; H = 2.50), depending on the metric, and CBPV (S = 27; H = 1.48). When comparing viral diversity between Vd+ and Vd- island groups (Fig 4), a positive effect of *Varroa* on ASV Richness (S) was observed for BQCV (Pr_(Vd+>Vd-)_ = 100%), LSV (Pr_(Vd+>Vd-)_ = 99.9%), and CBPV (Pr_(Vd+>Vd-)_ = 86.9%), as inferred by Bayesian modelling (Table 1). In contrast to S, the effect of *Varroa* on the Shannon-Wiener index H was only consistently detected for LSV (Pr_(Vd+>Vd-)_ = 98.7%). SBV was confined to Vd+ islands, meaning that comparisons of genetic diversity estimates between islands with a Vd+ and a Vd- status were not possible.

### Effect of *Varroa* on viral phylogeography

**Fig 5:**
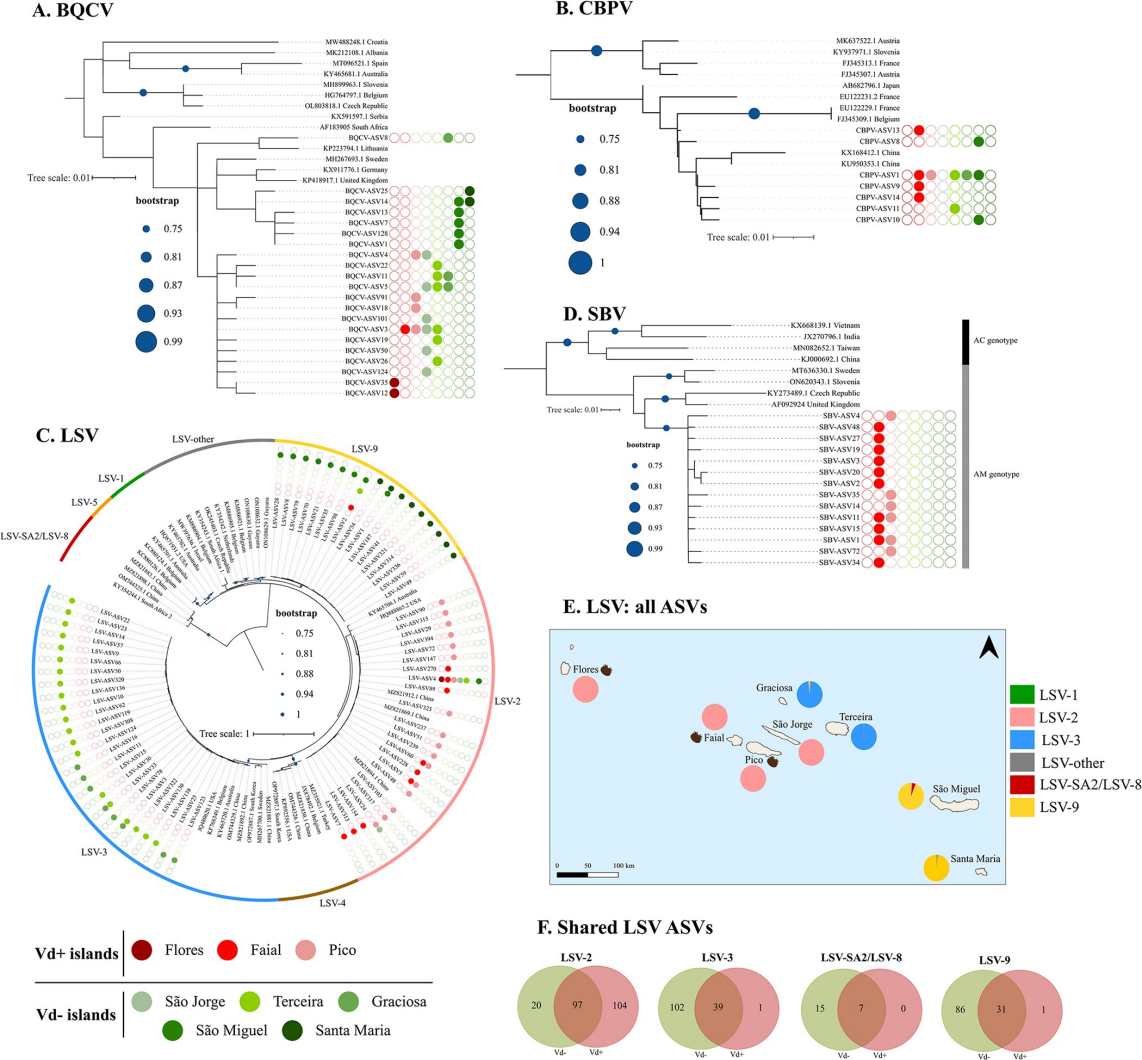
Phylogenetic reconstruction from the most abundant ASVs for (A) BQCV, (B) CBPV, (C) SBV, and (D) LSV in the Azores. The sequences generated in this study are indicated by a circle coloured according to the *Varroa* status: *Varroa*- invaded (Vd+) islands are marked by a red gradient whereas *Varroa*-free (Vd-) islands are marked by a green gradient. The filled/empty circles represent presence/absence of the viruses on the corresponding island. Sequences without the circles were retrieved from GenBank. The phylogenies were inferred from the maximum likelihood method using the Tamura 3-parameter model (bootstrap = 1000 replicates) for BQCV, and the Kimura-2 for SBV, CBPV, and LSV. (E) Geographic distribution of the LSV master variants identified from all ASVs. (F) Venn diagram showing the distribution of the number of all ASVs detected for each LSV master variant. The green circle represents Vd- islands, the red circle represents Vd+ islands, and the intersection represents the ASVs shared between the two island groups. Varroa islands are denoted by the varroa icon (created by www.biorender.com). The base map file of the Azores in Fig 5E was obtained from www.diva-gis.org.

A phylogeny was reconstructed for each virus using the most abundant ASVs (Fig 5). A shallow topology characterized by relatively short and poorly supported branches was recovered for most viruses. CBPV and SBV showed a rather geographically limited distribution, complicating identification of any structure shaped by *Varroa* or even geography, if present. Moreover, the unique ASVs of these two viruses, as well as the unique ASVs of the more widely distributed BQCV and LSV (Fig 5A–5D), were rarely shared among the islands, further complicating the detection of any effect of *Varroa* presence on the different islands on the viral phylogeographic patterns at the ASV level. However, upon closer examination of the phylogeographic patterns at the master variant level, a clear effect emerged for the multi-strain LSV (Fig 5C), which was only possible to detect due to the use of a RT-PCR assay designed specifically to identify multiple strains. The LSV ASVs grouped into three divergent and well-balanced clades, corresponding to three distinct LSV master variants, including LSV-3 (39.4%), LSV-2 (34.8%), and the novel LSV-9 (25.8%). It is plausible that LSV-9 evolved on the eastern islands of Santa Maria and São Miguel, where it was largely predominant, as shown by the spatial pattern retrieved from all detected ASVs (Fig 5E). LSV-9 was replaced by LSV-3 on Graciosa and Terceira and by LSV-2 on São Jorge and all Vd+ islands (Fig 5E). While the Vd- island São Jorge shared with all Vd+ islands the dominant LSV-2 master, a closer look at the distribution of the diversity of the LSV-2 ASVs shows that the number of unique ASVs was dramatically higher when *Varroa* was present (104 unique ASVs) than when it was absent (20 unique ASVs). Interestingly, this pattern was reversed for all other master variants, with only one unique ASV on the Vd+ islands for both LSV-3 and LSV-9 versus 102 unique LSV-3 ASVs and 86 unique LSV-9 ASVs on the Vd- islands (Fig 5F).

### Effect of *Varroa* on viral loads

**Fig 6:**
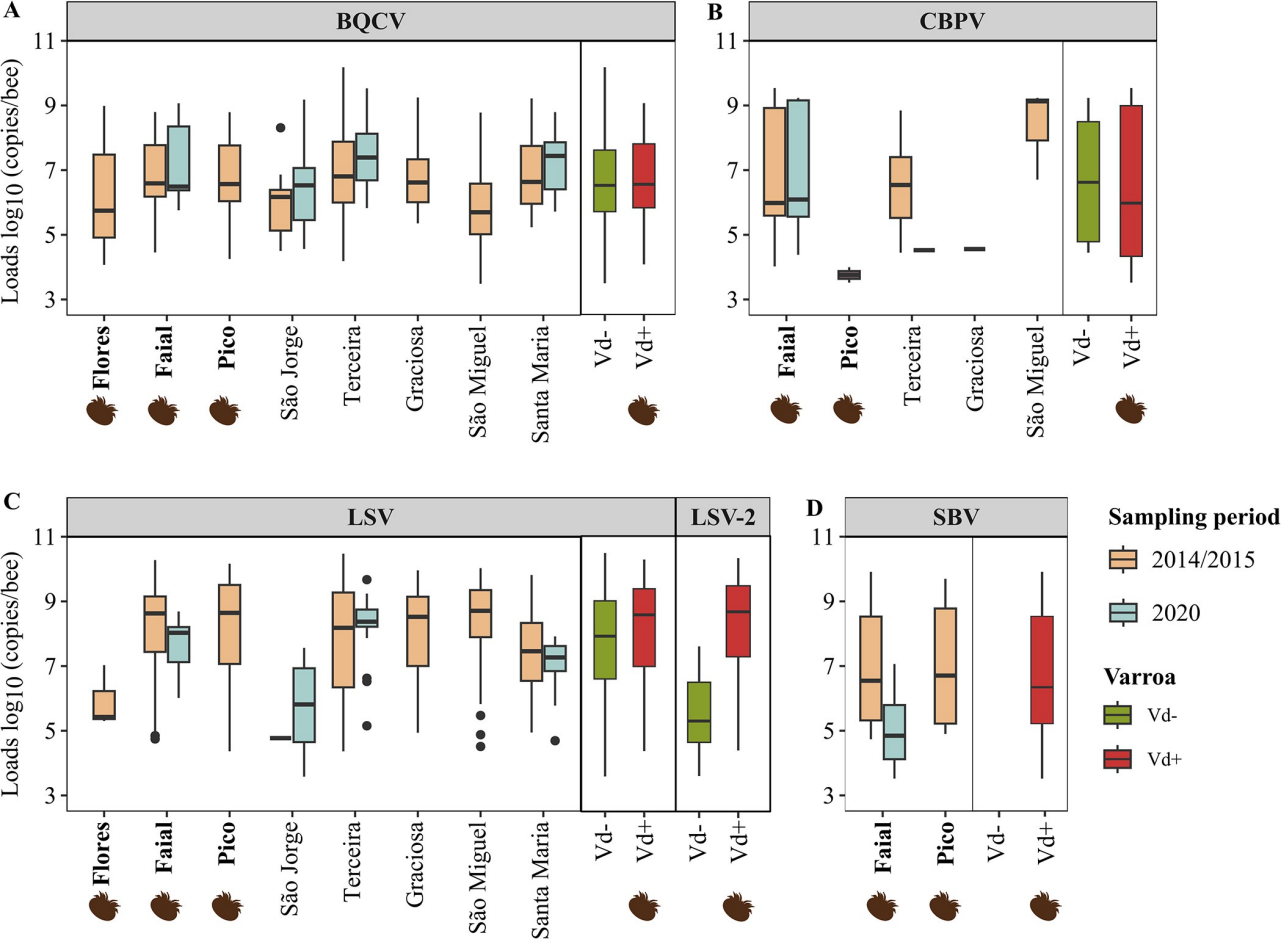
Boxplots of log_10_ viral loads (copies/bee) for each island and pooled samples from the *Varroa* -negative (Vd-, green bar) and *Varroa* -positive (Vd+, red bar) islands, by sampling period (2014/2015 and 2020). **(A)** BQCV- Black queen cell virus, **(B)** LSV- Lake Sinai virus, **(C)** CBPV- Chronic bee paralysis virus, **(D)** SBV- Sacbrood virus. Varroa-islands are denoted by the varroa icon (created by www.biorender.com).

Fig 6 illustrates the spectrum of virus loads observed for BQCV, LSV, CBPV, and SBV on each island and for both sampling periods (see S6 Table for detailed descriptive statistics). BQCV exhibited similar median loads and variation across all islands of the Azores, ranging from 5.70 (IQR = 1.56) log_10_ copies/bee on São Miguel to 7.44 (IQR = 1.45) log_10_ copies/bee on Santa Maria (2020 sampling). In contrast, the loads of LSV varied greatly among islands and showed a wider range, from a low of 4.77 log_10_ copies/bee for the single LSV-positive colony detected in 2014/2105 on São Jorge to a high of 8.71 (IQR = 1.45) log_10_ copies/bee on São Miguel, both of which are Vd- islands. The highest median load of the less common CBPV was also detected on São Miguel (9.13 log_10_ copies/bee; IQR = 1.26). On the Vd+ islands, the median CBPV load was considerably lower, both on Pico (3.76 log_10_ copies/bee, IQR = 0.24) and on Faial (5.98 log_10_ copies/bee; IQR = 3.33), with no CBPV detected on Flores. In contrast to the other viruses, SBV was detected only on Vd+ islands, with Pico (6.70 log_10_ copies/bee, IQR = 3.56) and Faial (6.55 log_10_ copies/bee, IQR = 3.21) harbouring similar loads in 2014/2015.

Loads were then compared between Vd+ and Vd- island groups for each virus (Fig 6). They varied greatly within each group, and although no clear distinction could be visualized for any virus, the Bayesian modelling was able to capture a reasonably high probability of a positive effect of *Varroa* on BQCV load (Pr_(Vd+>Vd-)_ = 90.8%) but not on CBPV and LSV loads (Pr_(Vd+>Vd-)_ < 71.1%; Table 1). The effect of *Varroa* on the loads of the multi-strain LSV was then re-analysed by also taking into consideration the LSV phylogeographic structure (Fig 5). LSV-2 was the overwhelmingly dominant master variant on Vd+ islands and on the Vd- island of São Jorge, and it occurred at very low frequency on the other Vd- islands. Hence, when the analysis was performed separately for different LSV strains, an effect of *Varroa* on LSV-2 load became evident (Fig 6) and was strongly supported by Bayesian modelling (Pr_(Vd+>Vd-)_ = 100%; Table 1).

## Discussion

Six Azorean islands are part of the rare *Varroa*-free refugia in the world [44,53,63], a distinction recently acknowledged by the European Union [64]. However, the Azores also includes three islands that were invaded by the deadly ectoparasitic mite *Varroa destructor* at different time points: Pico in 2000, Flores in 2001, and Faial in 2008 [65]. This island combination positions the Azores as an exceptional natural laboratory to study how the invasion of *Varroa* affects honey bee viral landscapes. While there is unambiguous evidence that *Varroa* efficiently transmits DWV [7,29] and by doing so increases its prevalence and load [6,7,29,43,45,47,66], we have little understanding of the role that the mite has played in shaping other viruses landscapes. In this study, we sought to address this challenge by surveying eight important viruses in a comprehensive honey bee collection from the Azores. Our survey revealed a rather unique and heterogenous viral landscape, with viruses occurring on every island (BQCV, LSV), viruses occurring on some of the islands (CBPV, SBV), and viruses that were not detected at all (BeeMLV and the AKI complex: ABPV, KBV, IAPV). This finding complements previous reports on the same colonies, which identified islands devoid of two of the most harmful honey bee pathogens: DWV (Terceira and São Jorge; [46]) and the Microsporidia *Nosema ceranae* (Santa Maria and Flores; [57]). While the virulence of BeeMLV is unknown [31], *N*. *ceranae* and members of the DWV and AKI complexes have been implicated in winter colony losses [40,41,67,68]. Therefore, the absence of *Varroa* and potentially all these harmful pathogens make the Azores a unique place for beekeeping.

Building on current knowledge [43,45–47], we predicted that the viral landscape of *Varroa*-invaded (Vd+) islands (Pico, Flores, and Faial) would differ from that of *Varroa*-free (Vd-) islands (São Miguel, Santa Maria, Terceira, São Jorge, and Graciosa) by featuring higher prevalence, higher co-prevalence, and higher loads for most viruses. Furthermore, we expected *Varroa* to modify viral diversity and phylogeographic viral patterns. It turned out that the epidemiological situation in the Azores was more complex than anticipated. The prevalence of SBV, LSV, and CBPV was higher on Vd+ than on Vd- islands, consistent with the DWV findings generated from the same colonies [46]. Other studies have not found an increase in prevalence for LSV, but they did for CBPV and SBV, suggesting an effect of *Varroa* on their inter-colonial dissemination [43,45,47]. Although these independent observations are interesting, unambiguous empirical and experimental evidence proving *Varroa* as a vector of CBPV and SBV is lacking [6,29,50,69]. Therefore, it is more likely that the mite acted as a facilitator of intercolonial dissemination of these viruses, possibly via worker bees robbing *Varroa*-collapsed infected colonies [70] or infected drifting bees entering virus-free colonies, which are better accepted by *Varroa*-infested colonies [71].

Interestingly, the elevated prevalence of CBPV and LSV was accompanied by an elevated number of variants (Richness) for these viruses on Vd+ islands. Viruses generate *de novo* variation by error-prone replication, which is subsequently modulated by natural selection and genetic drift [72,73]. This implies that the higher the infection levels (as expressed by prevalence or loads), the higher the frequency of mutational events, and the lower the chance of losing variation by genetic drift. These mechanisms could explain the observed increase in Richness for both viruses. However, *Varroa* can also mediate viral diversity loss by selectively transmitting advantageous strains, as reported for DWV in Hawaii [47], leading to a negative relationship between load and estimated diversity. Such observations may however be affected by the method used for estimating diversity, with indirect global methods (such as the melting curve analyses used by Martin and colleagues [47]) more likely to report a negative relationship, while more direct methods (such as ASV diversity) more likely to report a positive relationship [46]. While both viruses exhibited elevated Richness in the presence of *Varroa*, only LSV exhibited elevated Shannon-Wiener diversity, which also takes into account the Evenness of the distribution of this Richness. It is possible that selection and/or genetic drift acted to reduce CBPV variation, maintaining one or a few dominant variants in the population, whereas for LSV selection and mutation acted to maintain a more evenly distributed quasispecies of several moderately dominant variants supplemented by numerous minor variants generated by the high mutation rate typical of this virus [11,12].

SBV was detected only on Pico and Faial, explaining the 100% posterior probably of prevalence rise on Vd+ islands (and the impossibility of modelling of the *Varroa* effect on SBV diversity and load). At least three hypotheses can be invoked to explain the apparent absence of SBV on the other islands. One possibility is that SBV occurred at very low frequency and/or viral loads were below the detection threshold on Vd- islands, and the relatively high prevalence and loads observed on Pico and Faial could be attributed to *Varroa* facilitating SBV dissemination [43,45,47] and promoting virus infection by suppressing individual and colony immunity [30,74]. Another possibility is that the primer-based methodology used in this study failed to capture cryptic SBV variation on the SBV-negative islands. However, all the virus PCR assay primers were expressly designed to detect as broad a range of variants as possible [10], the two known major SBV strains (the Asian and European) and, due to the low evolutionary rate of SBV, it is unlikely that there are other strains circulating in the honey bee populations [75]. Alternatively, these islands are truly naïve to SBV, and the virus might have opportunistically accompanied the illegal imports that brought *Varroa* to Pico in 2000. Subsequently, in 2008, SBV could have dispersed to the nearby Faial by hitchhiking on swarms that also carried the mite [65] or could have been introduced later through authorized honey bee trading between *Varroa*-invaded islands. The putative absence of SBV on Flores suggests that the illegal queen import introducing *Varroa* in 2001 was free from SBV. This observation not only reinforces the last hypothesis but also lends support to the claim of two independent primary migration events of the mite into the Azores [46].

In contrast to the other viruses, the effect size of *Varroa* on BQCV prevalence was virtually non-existent (-0.04; 28.5% posterior probability). This lack of statistical support for a *Varroa-* BQCV association was expected because this virus was highly prevalent on every island, irrespective of *Varroa* status. Besides, notably, the lowest frequency of BQCV-infected colonies was found on the Vd+ island of Flores (78.4%), contrasting with Santa Maria, Terceira, Graciosa, and Faial, where it was detected in every single inspected colony. Whether *Varroa* is implicated in BQCV intercolonial dissemination is unclear [6], with others documenting both no effect [43] or a significant increase in prevalence following mite invasion [44,45].

*Varroa* also altered viral co-prevalence, with colonies from Vd+ islands exhibiting a higher frequency of colonies hosting >2 viruses than colonies from Vd- islands. Whether these co-infections originated from multiple individuals with distinct mono-infections, from single individuals with multiple infections, or a combination of both is unknown because the analysis was performed on pooled individuals. Regardless, the presence of *Varroa* seemingly promoted mixed-virus infections within colonies, as was also observed in New Zealand [43]. A honey bee colony contains thousands of individuals living together in close proximity in a homeostatic nest. Coupled to certain social behaviours (*e*.*g*., trophallaxis), such an environment greatly facilitates the proliferation of different viruses within the nest, a situation that can be aggravated in the presence of a potent virus-transmission and immune-debilitating agent such as *Varroa* [30,76]. Therefore, it is not surprising that multiple viruses are commonly found within colonies (co-prevalence; [77,78]) or within individuals (co-infections; [79,80]), and the health impact of these co-prevalent or co-infecting viruses is heightened by *Varroa*, despite the multiple individual and social immunity adaptations that honey bees have evolved to minimize the damage of epidemic diseases within such a high density, disease-favourable nest environment [21,81].

The virus load patterns did not align well with the prevalence patterns. BQCV was the sole virus for whose prevalence was not altered by *Varroa*. At the same time, BQCV was the sole virus for which the load was altered by *Varroa*. However, similar to the other viruses, the presence of *Varroa* led to elevated ASV Richness in colonies infected by BQCV, in which case the generation of *de novo* variation would be linked to increased loads as opposed to increased prevalence. Despite the high posterior probability (90.8%) of a *Varroa* effect on BQCV load, the size of this effect was negligible (0.18 ± 0.16 log_10_ copies/bee), which, together with the lack of a prevalence effect, indicates a rather weak *Varroa*-BQCV association. This finding contrasts with that of [45], who recently reported a significant *Varroa*-induced effect on both BQCV prevalence and load. BQCV is the most widespread and common honey bee virus in the world [6], with surveys from all continents reporting prevalence rates ranging from 0% in northern Italy and Cuba [77,82] to 100% in the US and Canada [83,84], with a mean global value of 52.8% ± 34.3% (data extracted from 33 references, including [85–90], and citations therein). In the Azores, prevalence was well above the great majority of these worldwide reports, most often reaching 100% even in the absence of *Varroa*. Besides, BQCV was able to develop loads associated with overt infections (≥ 10^8^ copies per bee [91]) in 55 colonies from Vd- islands without the assistance of *Varroa*. These findings suggest that BQCV does not need a vector to be efficiently disseminated among colonies or develop high infections, and it is extremely well-adapted to the environmental conditions of the Azores.

The most intriguing discovery in the honey bee viral landscape of the Azores was revealed by the phylogeographic reconstructions of the viruses, particularly for LSV (reported here) and for DWV (reported in detail elsewhere [46]). This approach allowed us to efficiently disentangle the effect of geographic structure from the effect of *Varroa* on both viruses, which proved crucial for uncovering an otherwise overlooked link with the mite. The LSV diversity largely grouped into three divergent clades, corresponding to the master variants LSV-9, LSV-3, and LSV-2. LSV-9 is a novel LSV variant discovered exclusively, so far, on the Azores in this study. LSV was dominant on the eastern islands of São Miguel and Santa Maria and occurred at very low frequency on the remaining islands. This is a notable finding because these islands also host a divergent BQCV diversity and, more importantly, are a also refuge for the rare DWV-C master strain [46]. Whether this putatively novel LSV strain was originally introduced with the honey bee colonies brought into the Azores by the settlers in the 16th century, with queen lines introduced from Italy and France for a breeding program implemented in the 1980’s, or with queens occasionally introduced by beekeepers from varying geographical origins prior to the honey bee importation ban is unknown [46,65]. Irrespective of the time and route of introduction, the phylogeographic structure retrieved from the sequence diversity of several bee viruses suggests that the viral populations of these two proximate eastern Azorean islands share a similar evolutionary history, facilitated by similar selective pressures (*e*.*g*., similar climate, beekeeping management, and absence of *Varroa*) and that is maintained despite gene flow via honey bee trade with the other islands, or import from outside the Azores.

LSV-3 was the dominant strain on the Vd- islands of Terceira and Graciosa, but it also occurred in negligible amounts elsewhere across the Azores. LSV-3 was first described in 2012 [58] in colonies in the USA and has since been reported in different countries, usually at a lower prevalence than the earlier discovered LSV-1 and LSV-2 [9,61,92]. Interestingly, in Slovenia, LSV-3 is the dominant strain [93] and the queens that were introduced onto Graciosa in the 1980’s for breeding purposes were possibly of C-lineage *A*. *m*. *carnica* ancestry [65], which is the native honey bee subspecies of Slovenia and other countries in the Balkan-Caucasia region. The descendants of these queens were then cross-bred with other queen lines on Pico, and the hybrids were distributed to beekeepers on the other islands, especially Terceira [65,94]. While we can only speculate on the origin of LSV-3 in the Azores, Graciosa and Terceira share a LSV landscape that is remarkably distinct from that of the other islands of the Central Group.

Finally, and most remarkable, LSV-2 was overwhelmingly dominant on Pico, Faial, and Flores, where *Varroa* is present. While this finding could suggest that *Varroa* is driving the LSV evolutionary change or strain displacement on these islands, the viral landscape of São Jorge was also dominated by LSV-2, where *Varroa* is absent. LSV-2 is one of the most prevalent LSV master variants in the world, and its extant range likely predates the global spread of *Varroa* [12,45,95]. Therefore, it is plausible that LSV-2 was historically introduced across the Azores, where it existed in sympatry with other strains, and its nearly fixation on São Jorge was driven by genetic drift, a leading evolutionary force in small isolated populations [73]. The observation of one colony on Terceira and two on São Miguel dominated by the variant LSV-2-ASV04 and the residual occurrence of this and other ASVs of LSV-2 ancestry on all islands support this hypothesis. Moreover, the variants ASV04 and ASV05 dominated the LSV-2 landscape on Pico and Faial, in contrast to São Jorge, where these ASVs were rare. On the other hand, the dominant LSV-2-ASV24 variant on São Jorge was hardly found on Pico, Faial, and Flores. Further support for the action of genetic drift (São Jorge) or possible varroa-driven selection (Pico and Faial) for LSV-2 comes from the observation that dominant ASVs were rarely shared among islands.

While the dominance of LSV-2 on the Vd+ islands could be explained by either genetic drift or selection, the patterns retrieved from prevalence, load, and diversity suggest that *Varroa* is also an important modulator of the LSV landscape in the Azores. *Varroa* influenced positively the LSV prevalence on Vd+ islands. Interestingly, this effect was determined by the high number of colonies hosting LSV-2, and these carried significantly higher loads than the LSV-2 colonies originating from the Vd- islands. Thus, although we could not detect a biologically meaningful effect of *Varroa* on the loads of LSV (71.1% posterior probability), the mite did show a strong positive effect on the loads of the LSV-2 strain (100% posterior probability). Elevated prevalence and load were accompanied by elevated diversity on Vd+ islands. Of note is the extraordinarily high number of unique LSV-2 variants on Vd+ islands as compared to Vd- islands (104 *versus* 20), whereas the opposite pattern was observed for the other LSV master strains (Fig 5F).

Taken together, these findings reinforce the hypothesis that *Varroa* is driving the LSV evolutionary change in the Azores, particularly for LSV-2. In a similar study comparing viral landscapes between regions with and without *Varroa*, [45] also found a (weak) association of the mite with LSV-2 but not with LSV-1. Moreover, LSV-2 has been recurrently isolated from colonies with poor health [9,58,61]. Whether our findings are due to LSV-2 being efficiently transmitted to honey bee adults during *Varroa*’s feeding process or to an opportunistic response to immune-suppressed honey bees infested with mites and infected with other viruses (co-prevalence also increased significantly on Vd+ islands), or both, is uncertain [96,97]. Yet, inferring from the high prevalence observed on Vd- islands, it is certain that LSV as a virus species does not require *Varroa* to be efficiently transmitted from one colony to another.

## Concluding remarks

This study greatly advances our current knowledge on *Varroa*-virus interactions, with a particular emphasis on the multi-strain LSV. For the first time, we report robust empirical evidence supporting the association between *Varroa* and LSV-2. Moreover, we found a putatively novel LSV strain (tentatively named LSV-9) existing in sympatry with the extremely rare DWV-C, and these two viral strains are dominant on the easternmost islands of the Azores [46]. Three methodological aspects were key to our unprecedented discoveries. The first was the strategic design of RT-qPCR assays capable of detecting multiple strains of a virus in single reactions, in contrast to other assays that generally use strain-specific primers [45,95,98]. This allowed the simultaneous detection of five known LSV strains and the discovery of new strain. The second was the use of meta-amplicon deep sequencing, in contrast to other studies that used high-resolution melting curve analyses [47]. This enabled the retrieval of the full genetic variation spectra in every single colony and, thus, a more accurate appreciation of the impact of *Varroa* on viral genetic diversity. The third was the employment of a phylogeographic approach. The integration of these aspects enabled us to unravel the diverse mechanisms influencing the intricate viral landscape in the Azores and proved essential in elucidating *Varroa*-virus associations that might have otherwise gone unnoticed.

## Materials and methods

### Sampling location and procedure

Honey bee samples (in-hive adult workers) were collected from across the Azores, which is an Atlantic archipelago located ~1400 km west of Portugal. It comprises nine volcanic islands clustered into three groups (Fig 2): the eastern group (Santa Maria and São Miguel), the central group (Terceira, Graciosa, São Jorge, Pico, Faial), and the western group (Corvo, Flores). Of the nine islands, six are devoid of *Varroa* (Santa Maria, São Miguel, Terceira, São Jorge, Graciosa, and Corvo), and three have been invaded by the mite at different time points, namely: Pico in 2000, Flores in 2001, and Faial in 2008 [65].

Colonies were sampled between July and August of 2014/2015 and 2020, with the help of the local veterinary services. This sampling period coincides with the seasonal peak of most screened viruses [6], therefore minimizing false negatives related to their temporal dynamics. In 2014/2015, a total of 474 colonies were sampled from 150 apiaries distributed across eight islands (Corvo did not have honey bees until 2016, when six colonies were introduced from Terceira), with the number of apiaries sampled on each island proportional to the number of colonies registered on each island in 2013 (see S1 Table in [65] for the census size per island). Of these, 402 colony samples (originating from 150 apiaries) contained the minimum 30 workers (sterile females) required for colony-level virus analyses [10], and were included in the current study (Fig 2). In 2020, a total of 92 colonies, originating from 34 apiaries, were sampled from four islands, including Faial, Terceira, São Jorge, and Santa Maria (Fig 2). Most often, three colonies were sampled per apiary in the two sampling periods (S1 Table). Workers were collected alive and directly placed into ventilated cardboard boxes (one per colony sample) supplied with candy, and stored at 4°C until they were shipped alive by airplane to mainland Portugal where they arrived at the processing laboratory within 24 hours after collecting the sample in the field. Upon arrival at the laboratory, the samples were immediately stored at -80°C until extraction of nucleic acids. Prior research has shown that virus titres in pooled honey bee samples are unaffected by live transport over 24 hours, relative to instant freezing in liquid nitrogen [99].

### Extraction of ribonucleic acids and cDNA synthesis

For each of the 494 samples included in the experiment, covering both sampling periods, the total ribonucleic acids were extracted from a pool of 30 workers using the Monarch Total RNA Miniprep kit (New England Biolabs Inc., Massachusetts, US). Prior to extraction, the sample pools were macerated following the procedures detailed in [46]. The concentrations of RNA extracts were measured using the Epoch Microplate Spectrophotometer instrument (Agilent-BioTek Instruments, California, US) with the Take3 micro-volume plate accessory and subsequently normalized to a concentration of 250 ng/μL. Synthesis of cDNA was performed using 1 μg of RNA in a 20 μL reaction following the manufacturer’s instructions of the iScript^TM^ cDNA Synthesis Kit (Biorad, California, US). The RNA and cDNA extracts were stored at -80°C and -20°C, respectively, until further analysis.

### Real-time qPCR

Detection and loads of BQCV, CBPV, SBV, LSV, BeeMLV, and AKI-complex were assessed using real-time quantitative PCR (qPCR). The qPCR reactions were performed on the QuantStudio 5 apparatus (Applied Biosystems, Massachusetts, US) using the SYBR Green chemistry. The thermal cycling protocol implemented for all the viruses followed the SYBR Green manufacturer instructions, namely: 95°C for 30 seconds and 40 cycles of: 95°C for 15 seconds and 60°C for 60 seconds. This protocol was slightly modified for BeeMLV, wherein thermal conditions were adjusted to 56°C for 20 seconds and 60°C for 30 seconds, deviating from the original 60°C for 60 seconds. Each qPCR reaction was carried out in a 10 μL total volume, containing 3 μL of the diluted cDNA (1:10), 5 μL of 2× iTaq Universal SYBR Green Supermix (Biorad, California, US), and 500 nM of each primer (S2 Table). The primer pair for the multi-strain LSV was designed to detect at least four master variants, including LSV-1, LSV-2, LSV-3, and LSV-4 [11]. Each sample was run in duplicates, and the qPCR 96-well plate included a standard curve and two non-template controls. The standard curves for viral analysis were established from recombinant plasmids acquired from gene synthesis services at NZYTech Genes & Enzymes Company, Lisbon, Portugal (S3 Table). The absolute quantification of each virus was calculated using a duplicate standard curve containing seven calibration points consisting of 10-fold dilutions of a reference standard specific for the assay in question and of known concentration, ranging from 10^1^ to 10^7^ copies/μL template. The specificity of the synthesised amplicons was checked by performing a melting curve protocol ranging from 65°C to 95°C (increments of 0.5°C s^-1^). The qPCR efficiency and correlation coefficient for each screened virus are summarised in S4 Table. All samples were deemed positive if they amplified before the last point of the standard curve, had an exponential rise on the amplification plot, and had a melting profile matching the melting temperature of the positive controls. The mean viral loads were converted into copy number per bee by multiplying the copy number per sample by various dilution factors. To confirm the existence of amplifiable cDNA in each sample, the housekeeping gene RPL8 of *A*. *mellifera* was screened prior to viral screening (mean Ct ± SD; 20.8 ± 0.2).

### High-throughput sequencing and bioinformatics analysis

The amplicon sequencing libraries were constructed for all 262 LSV-positive, 26 CBPV-positive, and 45 SBV-positive colony samples. Given the high number of BQCV-positive samples (475), the library was constructed for a subset of 174 randomly chosen colonies, with each apiary represented.

All libraries were prepared following a two-stage amplification process, which is fully described in [46]. Briefly, in the first stage, Illumina sequencing adaptors, expanded by one to four random nucleotides to enhance the sequencing read quality [100], were added to the initial PCR (see primers and adapters in S2 Table). Six positive samples of LSV and two of CBPV failed amplification and were not further analysed. In the second stage, the amplicons were subjected to indexation PCR, which consisted of the incorporation of the P5 and P7 Illumina-specific adapters. The indexed amplicons underwent purification, quality control, quantification, normalization to 20 nM, and pooling. Each viral library pool was assessed for amplicon size distribution using the D5000 Kit in the TapeStation 220 (Agilent Technologies Inc., California, US) and quantified using the SYBR green qPCR assay (KAPA Library Quantification Kit, Kapa Biosystems, Massachusetts, US) to be subsequently pooled in a single library. Following denaturation and recommended dilution by Illumina, the pooled viral library was loaded at 12 pM on a MiSeq flow cell with 10% PhiX spiked in. The libraries were then sequenced on the Illumina MiSeq platform (Illumina Inc., California, US) using the 2 × 250 cycle v2 chemistry, following the manufacturer’s instructions.

The sequencing reads were demultiplexed in the Illumina BaseSpace Sequence Hub based on the unique indexes integrated during library preparation, which generated two FASTQ files per sample (R1 and R2). These files were transferred to the Galaxy platform (usegalaxy.org; [101]) and processed using Mothur [102], in accordance with the standardized procedure for MiSeq data analysis [103–105] with minor modifications (for a detailed description of the bioinformatics pipeline, see [46]). S5 Table provides a comprehensive summary of key sequencing metrics obtained for each viral library, including the total count of sequencing reads, the number of poor-quality reads, denoising/chimeric reads, the overall read count after completion of the filtering procedures, and the median read count per sample. Following all filtering procedures, only unique sequences with more than five reads were retained.

### Phylogeny and diversity measures

The phylogenetic trees were constructed for each virus using the single most abundant ASV identified in each colony sample. To that end, these most abundant ASVs (one ASV per colony sample) were aligned with reference sequences retrieved from GenBank using the Clustal*W* algorithm, and the phylogenetic relationships were inferred using the Maximum Likelihood method within MEGA v10.0 [106]. The best-fit nucleotide substitution model was determined for each virus dataset using the Akaike Information Criteria (AIC) in MEGA. The selected model was Tamura 3-parameter [107] for BQCV and Kimura 2-parameter [108] for LSV, CBPV, and SBV. Node support was assessed using 1000 bootstrap replicates to evaluate the robustness of the phylogenetic tree topology. The resulting phylogenetic trees were further annotated and edited on the Interactive Tree of Life (iTOL) [109]. The GenBank accession numbers of the reference sequences used are shown in the phylograms, together with their country of origin. For SBV there is a further distinction between those sequences first recovered from *Apis mellifera* (AM) and those first recovered from *Apis cerana* (AC), where sacbrood is a major and lethal disease [110].

Diversity was assessed for each virus and colony sample by means of Richness (S), which reflects the number of different ASVs in the sample, and Shannon-Wiener index (H), which combines Richness and Evenness (the relative abundance of each ASV). These diversity metrics were calculated from the total ASVs detected for each virus using the ’vegan’ package [111] in R software [112].

### Statistical analysis

Apparent prevalence (with 95% confidence intervals, CI) was calculated using the *epi*.*prev* function from the ‘epiR’ [113], and the viral loads were log_10_ transformed for all graphic representations and analyses in R software [112]. A generalized linear mixed model (GLMM) approach was used to estimate the size effects of *Varroa* on prevalence (logit-link Bernoulli), co-prevalence (prevalence: logit-link Bernouli; Nr. viruses: Poisson), loads (logNormal), and diversity (Richness: Poisson; H’: Gamma) of each detected virus. In these models, *Varroa* status (presence; Vd+; absence: Vd-) and sampling year were set as fixed effects, and the apiary was included as a group-level random effect on the intercept (see S1 Appendix). *Varroa* infestation at the colony level was not considered in the analysis because the sampling was performed following the seasonal miticide treatment, so *Varroa* counts might not be an accurate indicator of colony infestation. The diversity and load models took into account only the viruses that were detected on both Vd+ and Vd- islands. Modelling was implemented in a Bayesian framework using JAGS [114] called from R [112]. For all models, minimally-informative priors were used, and the Monte Carlo Markov chains for 10,000 iterations were sampled after convergence had been reached, as determined by visual inspection of stability and mixing. Posterior predictive checks were used to assess model fit.

## Figures and licensing

All figures were our own work except for the Azores base maps in Fig 2 and Fig 5 and the varroa icon used in Figs 1, 2, 4, 5 and 6. The Azores location and topographical maps were constructed with the open-source geographic information system software QGis 3.22 (https://qgis.org) using a base map file downloaded from DIVA-GIS (www.diva-gis.org), both released under the GNU General Public License (GPL). The varroa icon was created at BioRender.com and was downloaded under a student/postdoc license, which permits use and publication in scientific journals. The text and images created by the authors were superimposed on the maps, for illustrative purposes.

## Supporting information

S1 Table Metadata of the sampled apiaries. (XLSX)

S2 Table Primers used in (a) real time-qPCR quantification and (b) high-throughput sequencing, and Illumina adapters. For the preparation of high-throughput sequencing libraries, new forward and reverse oligos were created for each virus using the following construction: 5’-Illumina adapter + 1 to 4 random nucleotide (N) + virus primer -3’. BQCV- Black queen cell virus; LSV- Lake Sinai virus; CBPV- Chronic bee paralysis virus; SBV- Sacbrood virus; BeeMLV-Bee macula-like virus; AKI–Acute bee paralysis virus, Kashmir bee virus and Israeli acute paralysis virus. The LSV primer pairs (#) are consensual for LSV-1, LSV-2, LSV-3 and LSV-4. The references for the assays are as follows: 1 = Locke *et al*. (2012) Acaricide treatment affects viral dynamics in Varroa destructor-infested honey bee colonies via both host physiology and mite control. Applied and Environmental Microbiology 78: 227–235. 2 = Daughenbaugh *et al*. (2015) Honey bee infecting Lake Sinai viruses. Viruses 7: 3285–3309. 3 = de Miranda *et al*. (2015) Genome characterization, prevalence and distribution of a Macula-Like virus from *Apis mellifera* and *Varroa destructor*. Viruses 7: 3586–3602. 4 = Evans (2006) Beepath: An ordered quantitative-PCR array for exploring honey bee immunity and disease. Journal of Invertebrate Pathology 93: 135–139. 5 = Mondet *et al*. (2014) On the front line: Quantitative virus dynamics in honeybee (*Apis mellifera* L.) colonies along a new expansion front of the parasite *Varroa destructor*. PLoS Pathogens 10: e15.
(XLSX)

S3 Table Recombinant plasmids used to generate qPCR standard curves BQCV- Black queen cell virus; LSV- Lake Sinai virus; CBPV- Chronic bee paralysis virus; SBV- Sacbrood virus; BeeMLV-Bee macula-like virus. (XLSX)

S4 Table qPCR performance for each screened virus. BQCV- Black queen cell virus; LSV- Lake Sinai virus; CBPV- Chronic bee paralysis virus; SBV- Sacbrood virus; BeeMLV-Bee macula-like virus; AKI–Acute bee paralysis virus, Kashmir bee virus and Israeli acute paralysis virus. No performance parameters are given for the AKI complex (*), because all Azorean samples were negative for the AKI complex assay.
(XLSX)

S5 Table Metrics of the high-throughput sequencing run. BQCV- Black queen cell virus; LSV- Lake Sinai virus; CBPV- Chronic bee paralysis virus; SBV- Sacbrood virus.
(XLSX)

S6 Table Descriptive statistics of the loads (log_10_ [copies virus/bee]) obtained for each virus by sampling period and island: median, interquartil range (IQR), minimum (Min), and maximum (Max). BQCV- Black queen cell virus; LSV- Lake Sinai virus; CBPV- Chronic bee paralysis virus; SBV- Sacbrood virus. *V*. *destructor* islands are represented in bold; “-”denotes virus not detected; * denotes only one positive colony.
(XLSX)

S1 Appendix Formal descriptions for the Bayesian statistical models used in the analyses. (DOCX)
