# Supplementary material for: Varroa destructor shapes the unique viral landscapes of the honey bee populations of the Azores archipelago
Source: PLoS Pathog. 2024 Jul 3;20(7):e1012337. doi: 10.1371/journal.ppat.1012337 (PMC11221739; doi:10.1371/journal.ppat.1012337)
Supplement: S1 Appendix — (DOCX) [file ppat.1012337.s007.docx]

**S1 Appendix**

These are the formal model descriptions for the Bayesian statistical modelling:

**1. Model relating viruses prevalence (as the response variable) to *V. destructor*.** In the Eq.1, ***virus*** (BQCV, LSV, CBPV, and SBV) was determined to be present or absent in a colony. We used a Bernoulli (i.e., a 1/0 binomial) distribution with a logit-link to estimate the importance of *V. destuctor* island status (varroa) and sampling year (year). Because there were colonies sampled from the same apiary, we included ‘apiary’ as a random effect on the intercept to adjust for local factors that might influence the probability of viral infection. The index ‘i’ indicates individual colonies sampled for each of the viruses, and the index ‘j’ indicates the number of sampled apiaries. The Bernoulli parameter ‘p’ is the estimated prevalence of each pathogen.

| Hierarchical model structure |
| --- |
| *V. destructor* effect (Eq. 1) |
| *virus* presence_i_ ~ Bernoulli (p_i_)  logit(p_i_) = intercept _j_ + b1*varroa_i_ + b2*year_i_  intercept _j_ ~ Normal (mu, sigma)  Priors  mu~Normal(0, 100)  sigma~Uniform(0,5)  b1~Normal(0,100)  b2~Normal(0,100) |

**2. Model relating viral load (log-transformed as the response variable) to *V. destructor* (Eq. 2)** Here, we restricted the dataset only to colonies that were positive for each of the ***virus*** (BQCV, LSV, CBPV, SBV, i.e., excluded all measures of zero from the analysis; thus this analysis is independent of the prevalence analysis in Eq.1). Viral load was a highly-skewed continuous variable and the log-transformation of data normalised the response variable (thus making the analysis a LogNormal model). Here, we used the same basic model structure as used above in Eq. 1, relating viral load to *V. destructor* island status (varroa) and year of sampling (year), with apiary included as a random effect in the intercept. Because there were colonies sampled from the same apiary, we included ‘apiary’ as a random effect on the intercept to adjust for local factors that might influence the probability of viral infection. The index ‘i’ indicates individual colony samples for each viruses load and the index ‘j’ indicates the number of sampled apiaries.

| Hierarchical model structure |
| --- |
| *V. destructor* effect (Eq. 2) |
| *virus* load_i_ ~ Normal (mu_i,_ sigma)  mu_i_ = intercept _j_ + b1*varroa_i_ + b2*year_i_  intercept_j_ ~ Normal (mu2, sigma2)  Priors  mu~Normal(0, 100)  sigma~Uniform(0,5)  mu2~Normal(0, 100)  sigma2~Uniform(0,5)  b1~Normal(0,100)  b2~Normal(0,100) |

**3. Model relating viral co-infection to *V. destructor*.** Here, we restricted the dataset only to colonies that were positive for at least 2 virus. We used the same basic model structure as used above in Eq. 1 and 2, relating co-infection to *V. destructor* island status (varroa) and year of sampling (year), with apiary included as a random effect in the intercept. Because there were colonies sampled from the same apiary, we included ‘apiary’ as a random effect on the intercept to adjust for local factors that might influence the probability of viral infection. The index ‘i’ indicates individual colony samples for each viruses load and the index ‘j’ indicates the number of sampled apiaries.

**Hierarchical model structure**

| *V. destructor effect* – Co-infections (Eq.3) |
| --- |
| coinfections_i_ ~ Poisson (lambda_i_)  log(lambda_i_) = intercept_j_ + b1*varroa_i_ + b2*year_i_  intercept_j_ ~ Normal (mu, sigma)  Priors  mu~Normal(0, 100)  sigma~Uniform(0,5)  b1~Normal(0,100)  b2~Normal(0,100) |

**4. Models relating measures of virus diversity (i.e. Richness (Eq.4) and Shannon-Wiener index (Eq.5)) to *V. destructor*.** Here we restricted the dataset only to colonies that were positive for each of the virus (LSV, BQCV, and CBPV) (i.e. excluded all measures of zero from the analysis). Richness was a count of virus variants, and thus we used a Poisson distribution to describe them (with a log-link), while Shannon-Wiener were positive continuous data and so we used a Gamma distribution to describe these. Again we used the same basic model structure as used above in Eq. 1 relating each diversity measure to *V. destructor* island status (varroa) and year of sampling (year), with apiary included as a random effect in the intercept. The index ‘i’ indicates individual colony samples for virus diversity, and the index ‘j’ indicates the number of apiaries sampled.

| Hierarchical model structure | |
| --- | --- |
| *V. destructor effect* – Richness (Eq.4) | ***V. desctructor effect* – Shannon-Wiener index (Eq.5)** |
| richness_i_ ~ Poisson (lambda_i_)  log(lambda_i_) = intercept_j_ + b1*varroa_i_ + b2*year_i_  intercept_j_ ~ Normal (mu, sigma)  Priors  mu~Normal(0, 100)  sigma~Uniform(0,5)  b1~Normal(0,100)  b2~Normal(0,100) | shannon_i_ ~ Gamma (a_i,_ b_i_)  mu_i_ = intercept_j_ + b1*varroa_i_ + b2*year_i_  intercept_j_ ~ Normal (mu2, sigma2)  a_i_ = mu_i_ ^2 / sigma^2  b_i_ = mu_i_ / sigma^2  **Priors**  mu~Normal(0, 100)  mu2~Normal(0, 100)  sigma~Uniform(0,5)  sigma2~Uniform(0,5)  b1~Normal(0,100)  b2~Normal(0,100) |
